# Spontaneous periodic ordering on the surface and in the bulk of dielectrics irradiated by ultrafast laser: a shared electromagnetic origin

**DOI:** 10.1038/s41598-017-12502-4

**Published:** 2017-09-26

**Authors:** Anton Rudenko, Jean-Philippe Colombier, Sandra Höhm, Arkadi Rosenfeld, Jörg Krüger, Jörn Bonse, Tatiana E. Itina

**Affiliations:** 1 Univ Lyon, UJM-St-Etienne, Laboratoire Hubert Curien, CNRS UMR 5516, F-42000 Saint-Etienne, France; 2 Max-Born-Institut für Nichtlineare Optik und Kurzzeitspektroskopie (MBI), Max-Born-Strabe 2A, D-12489 Berlin, Germany; 3 Bundesanstalt für Materialforschung und –prüfung (BAM), Unter den Eichen 87, D-12205 Berlin, Germany; 4 ITMO University, Kronverskiy pr. 49, St. Petersburg, Russia

## Abstract

Periodic self-organization of matter beyond the diffraction limit is a puzzling phenomenon, typical both for surface and bulk ultrashort laser processing. Here we compare the mechanisms of periodic nanostructure formation on the surface and in the bulk of fused silica. We show that volume nanogratings and surface nanoripples having subwavelength periodicity and oriented perpendicular to the laser polarization share the same electromagnetic origin. The nanostructure orientation is defined by the near-field local enhancement in the vicinity of the inhomogeneous scattering centers. The periodicity is attributed to the coherent superposition of the waves scattered at inhomogeneities. Numerical calculations also support the multipulse accumulation nature of nanogratings formation on the surface and inside fused silica. Laser surface processing by multiple laser pulses promotes the transition from the high spatial frequency perpendicularly oriented nanoripples to the low spatial frequency ripples, parallel or perpendicular to the laser polarization. The latter structures also share the electromagnetic origin, but are related to the incident field interference with the scattered far-field of rough non-metallic or transiently metallic surfaces. The characteristic ripple appearances are predicted by combined electromagnetic and thermo-mechanical approaches and supported by SEM images of the final surface morphology and by time-resolved pump-probe diffraction measurements.

## Introduction

The ability to inscribe periodic nanostructures on the surface and in the bulk of dielectrics by ultrafast laser irradiation has been a subject of increasing interest and active investigation during few last decades^1^. Not surprisingly, the polarization-dependent, rewritable and the smallest structures ever created by light are promising in optical data storage, security color marking, tribology, nanofluidics and computer holography^1–4^. Although the mechanisms of their formation are controversially discussed and are not clearly distinguished^1,5–7^, there is a strong experimental evidence of the electromagnetic nature of the phenomena due to local field polarization, incident angle dependency of the nanostructure orientation^3,8–11^, and laser wavelength dependency of the nanostructure periodicity^1,12^. The key role of inhomogeneous scattering centers attributed to initial surface nanoroughness, laser-induced defects, pre-distributed grooves, nanovoids or nanopores in case of bulk nanostructuring was also evidenced in experiments^7,13–18^ and proven numerically^19–23^.

In spite of the fact that laser-induced periodic surface structures (LIPSS) and volume nanogratings have many characteristics in common, both are often attributed to different phenomena, taking place close to surface or deep in the bulk of the material^5^. On one hand, volume nanogratings have striking similarities with femtosecond high spatial frequency LIPSS (HSFL), sharing sub-wavelength periodicity as well as laser polarization and laser wavelength dependencies^24^. Additionally, the transition between the surface ripples and the volume nanogratings was experimentally observed^25,26^. A closer examination revealed that the periodically arranged nanoplanes were preferentially formed at the interface between the regions affected and unaffected by the femtosecond laser irradiation^27^. It was then proposed that the mechanisms of the nanostructure formation were related^26–28^, but this has never been demonstrated. On the other hand, the formation of volume nanogratings was observed only in few glasses^29–31^. Additionally, higher number of applied pulses are required and the nanostructures are formed only within a certain narrow laser parameter window^2,12^.

Besides the HSFL structures, oriented perpendicular to the laser polarization, low spatial frequency ripples parallel to the laser polarization (LSFL-||) are typically formed for higher fluences or number of pulses on silica-based glasses^32–35^. Their formation mechanism was explained by Sipe theory representing an analytical solution for electromagnetic wave interaction with rough surface^36^ and further expanded by taking into account the changes of the optical properties of fused silica during ultrashort laser irradiation^33^. Since the formation of the HSFL structures cannot be explained within this approach, the transition from HSFL to LSFL-|| structures has never been investigated in the frame of a numerical approach.

Sipe’s theory also predicted the formation of low spatial frequency perpendicularly oriented ripples (LSFL-⊥) on the surface with metallic properties^36^. Although these structures are barely observed in fused silica^37^, they are often reported for low band gap dielectric and semiconductor materials, which easily turn metallic by ultrashort laser-induced excitation^7,8,38^. The different orientation between LSFL-|| and LSFL-⊥ for altering optical properties of the material (non-metallic and metallic) was supported by a series of the electromagnetic simulations based on the three-dimensional Maxwell’s equations^19^. However, neither the nonlinear excitation processes nor the Gaussian spatio-temporal shape of the laser irradiation source were taken into account. Furthermore, the transition from HSFL to LSFL-⊥ structures, observed in several independent experiments^8,13,39,40^, has never been explained and investigated numerically.

In this article, the formation mechanisms of several types of LIPSS (LSFL-||, LSFL-⊥ and HSFL), commonly observed on the surface of dielectrics^32–35^, are elucidated. The numerical results are supported by *ex-situ* surface imaging and by *in-situ* time-resolved pump-probe diffraction experiments. Furthermore, we show that volume nanogratings and the HSFL nanoripples are formed by the same electromagnetic mechanism of local field enhancement having different initial roots (surface roughness or nanopores in the bulk).

For this, we examine ultrashort laser interactions with a rough surface and the bulk containing randomly distributed inhomogeneities. In the proposed method, Maxwell’s equations are coupled with a rate equation, taking into account the ionization mechanisms and the transient changes of fused silica’s optical properties. Therefore, this self-consistent approach allows us to investigate directly the dynamics of the electronic modification in a more appropriate way than the linear electromagnetic or the analytic Sipe-theory based approaches^19,22,35^. In order to take into account the multipulse accumulation effects, a feedback mechanism providing the void-like structure growth into the nanogratings based on the near-field enhancements is proposed.

Additionally, a multiphysical model is developed, combining the nonlinear Maxwell’s equations and rate equation with the electron-ion heat transfer and thermoelastic wave equations, as well as Grady’s criterion for spall in liquid^41^ and hydrodynamic Rayleigh-Plesset equations^42^. The numerical results allow us to explain the ultrashort laser-induced mixed ripple morphologies and to interpret the experimental time-resolved diffraction measurements.

## Results and Discussion

### Formation mechanisms

**Figure 1:**
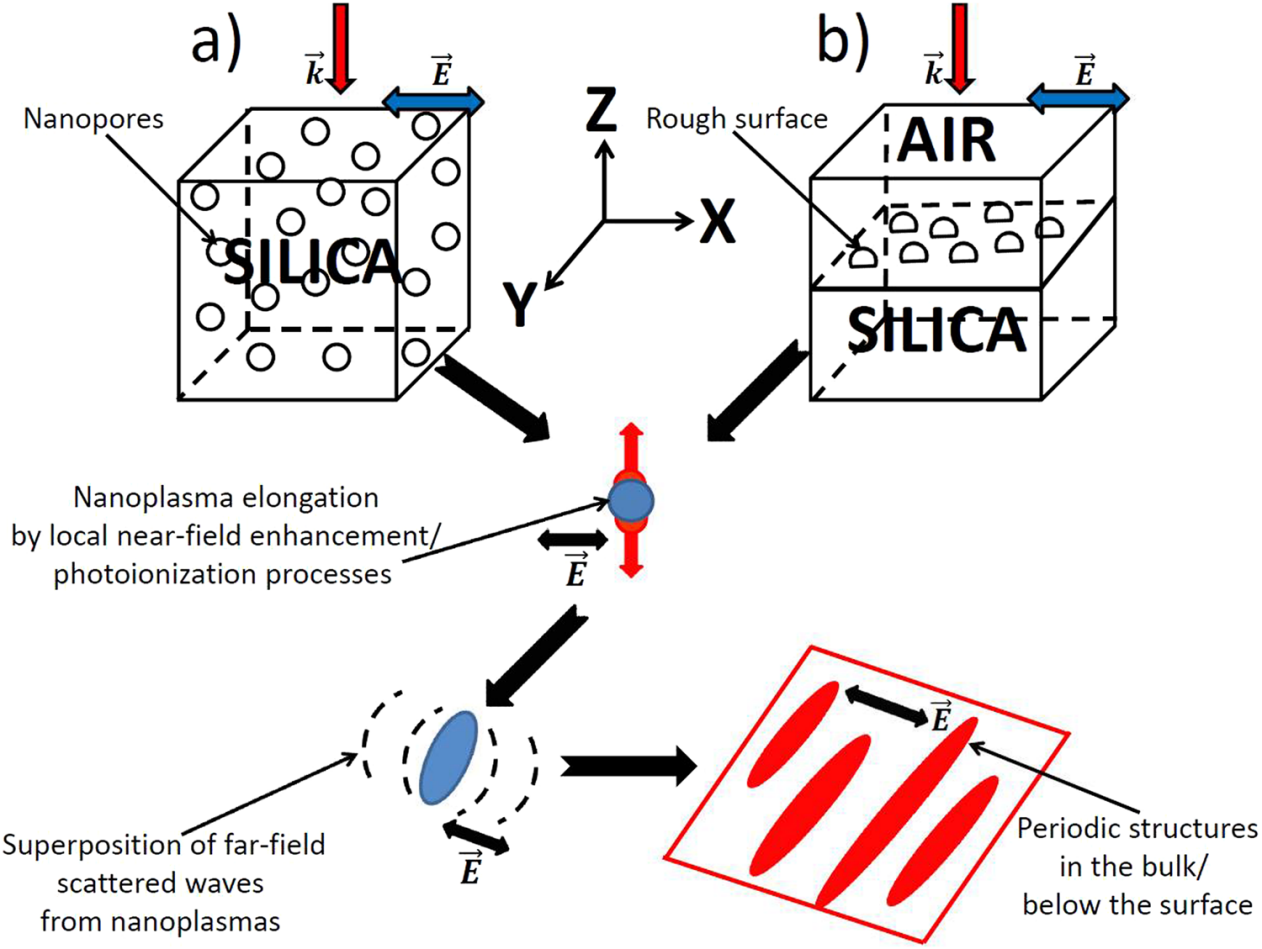
Schematic representation of the electromagnetic formation mechanisms of periodic nanostructures for (**a**) bulk and (**b**) surface ultrashort laser nanoprocessing.

The electromagnetic scenarios of subwavelength nanostructure formation on the surface and in the bulk of glasses are illustrated in Fig. 1.

**Figure 2:**
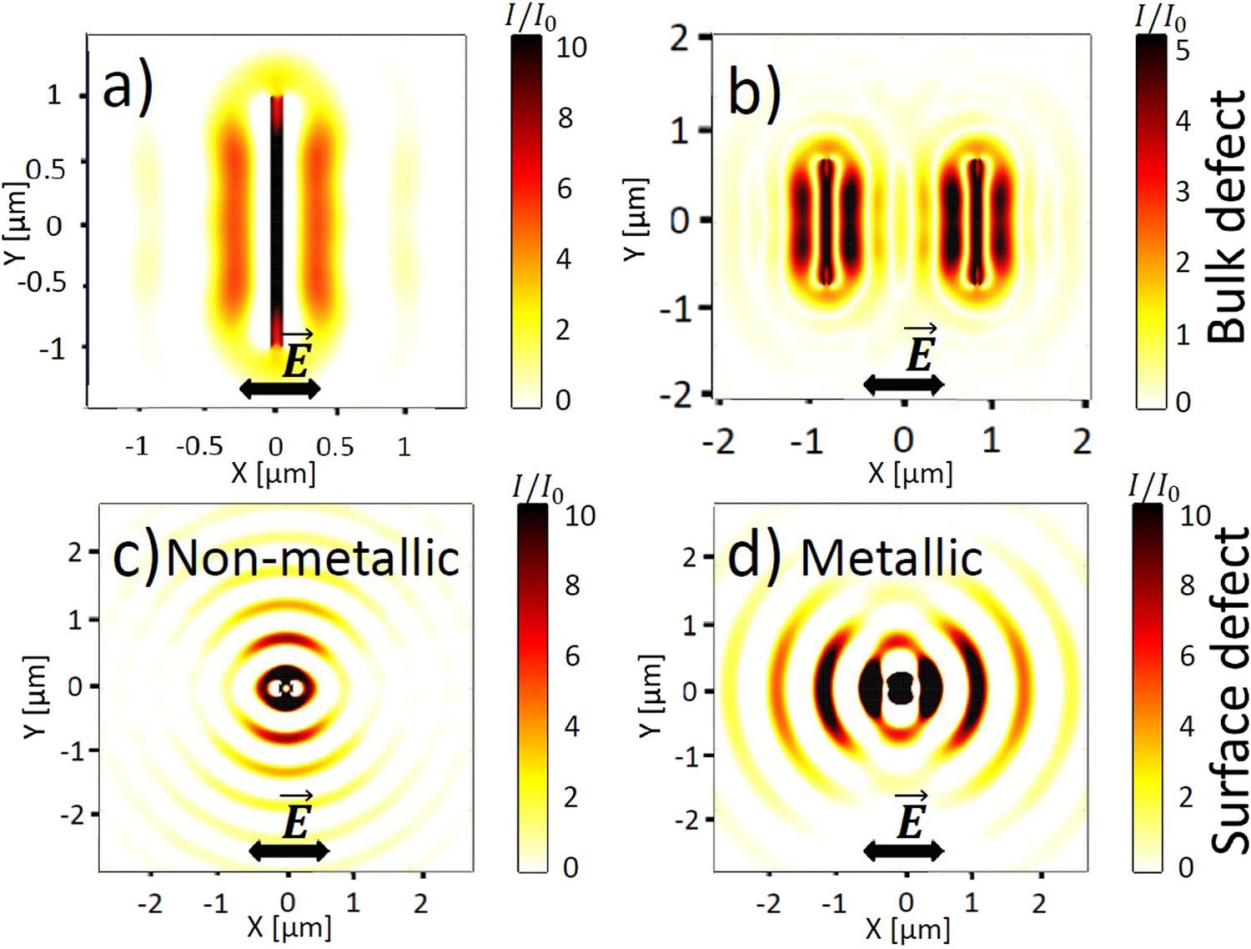
Bulk defect: (**a**) Intensity distribution integrated over the period around a single void nanoplane ${L}_{x}=80$ nm, ${L}_{y}=2$ μm, ${L}_{z}=500$ nm. (**b**) Intensity distribution integrated over the period as a result of the coherent superposition of the scattered waves from two void nanoplanes ${L}_{x}=80$ nm, ${L}_{y}=1.5$ μm, ${L}_{z}=50$ nm, separated by 2 μm in x-direction. Surface defect: Intensity pattern integrated over the period from a single inhomogeneity (hemisphere with $R=50$ nm) (**c**) on the non-excited fused silica surface ($\varepsilon =2.105$), (**d**) on the excited metallic fused silica surface ($\varepsilon =-1+0.5\cdot i$). Laser wavelength is fixed to be $\lambda =800$ nm.

In the case of bulk nanostructuring (Fig. 1a), the laser-induced nanopores/nanovoids with a radius $r=10$ nm, reported in several experimental articles^31,43^, are considered as the scattering centers. During ultrashort laser pulse irradiation, ionization processes reinforced by local field enhancement in the vicinity of the scattering centers lead to the generation of localized hot spots of higher electron densities or nanoplasmas. Upon material relaxation, these nanoplasmas can then turn into void-like structures before the next pulse interacts with glass. Local field enhancement contributes to their growth into nanoplanes in the direction perpendicular to the laser polarization on a shot-to-shot basis^2,43,44^. In addition to the near-field enhancement, each nanoplasma or inhomogeneity center scatters spherical waves into the far-field, which are enhanced parallel to the nanoplane. The intensity enhancement is getting stronger as the nanoplasmas elongate deeper below the irradiated surface^45,46^. Figure 2(a) shows the intensity distribution from an ideal single elongated void nanoplane. The interference of the incident field with the scattered field results in the periodic modulation with laser wavelength in media periodicity perpendicular to $\vec{E}$. If several scattering centers are involved, the coherent superposition of the multiple scattered waves results in the subwavelength periodicity as shown in Fig. 2(b). In general case, the final periodicity of the nanostructures is related to the inhomogeneity concentration *C* or, equivalently, the average distance between the nanoplasmas ${\rm{\Delta }}R$ ^23^. The concentration of the inhomogeneities at the fused silica-air interface here is defined as $C=N\pi {r}^{2}/(\pi {w}_{0}^{2})$, where *N* is the number of inhomogeneities of the characteristic radius *r* in the laser-induced area $S=\pi {w}_{0}^{2}$. The corresponding average distance is ${\rm{\Delta }}R=\sqrt{\pi {r}^{2}/C}$.

In the case of surface nanostructuring, the rough surface between air (dielectric permittivity $\varepsilon =1$) and fused silica with half-sphere inhomogeneities of the same radius $r=10$ nm is considered in Fig. 1(b). No scattering centers are introduced inside fused silica. Below the surface, random perpendicular oriented patterns are generated by the interference of the incident field with near-field scattered waves by single inhomogeneities, so-called roughness-dependent radiation remnants^19,22^. These patterns are the seeds for the periodic HSFL formation. Note, that small visible randomly-distributed cracks perpendicularly oriented to $\vec{E}$ are often reported in the experimental literature for low number of applied pulses^14,18,47,48^. The following scenario is similar to the one of volume nanogratings formation, as the nanoplasmas grow from the seeds due to the local field enhancement and the superposition of waves scattered at inhomogeneities results in periodic modulation perpendicular to $\vec{E}$. We emphasize that the presence of the air-fused silica interface is not necessary for the HSFL structures or volume nanogratings formation^23^. We note also that neither the local field enhancement nor the interference of the scattered waves require metallic optical properties for the excited glass matrix, however, the growth of the nanoplasmas can be significantly accelerated by the ionization processes in dielectrics if the pre-distributed inhomogeneous seeds for the nanostructure formation gained the metallic properties^23^.

Apart from its role in near-field enhancement of the incident light, the rough surface is the origin of the far-field periodic modulation, enhanced in parallel direction to the electric field vector $\vec{E}$ for non-metallic surface with $Re(\varepsilon ) > 0$ and in perpendicular direction for metallic surface with $Re(\varepsilon ) < 0$. The typical intensity distributions from one single void hemisphere $\varepsilon =1$ on the non-excited fused silica surface and on the metallic fused silica surface are shown in Fig. 2(c) and in Fig. 2(d) respectively. The dominant intensity patterns enhancement in the directions perpendicular (Fig. 2c) and parallel (Fig. 2d) to $\vec{E}$ is clearly seen and was firstly explained by Sipe’s theory^36^ and then investigated by FDTD simulations to address the orientation and periods of the intensity patterns at larger depths in the sample^19^. We note here, that the presence of the interface (surface) is essential for generation of the far-field periodic intensity patterns, as it confines the scattering centers in a joint plane. Furthermore, we underline, that the condition $Re(\varepsilon ) < 0$ is sufficient to generate the perpendicular oriented patterns, even if the surface plasmon wave excitation condition, $Re(\varepsilon ) < -1$, is not satisfied.

In what follows, we investigate the mechanisms of ripple formation on fused silica surface on the basis of electron density patterns calculated by the recently developed self-consistent approach^49^, where Maxwell’s equations are coupled to a free carrier rate equation taking into account the ultrafast transient changes for fs-laser irradiated fused silica.

### Surface nanostructuring

**Figure 3:**
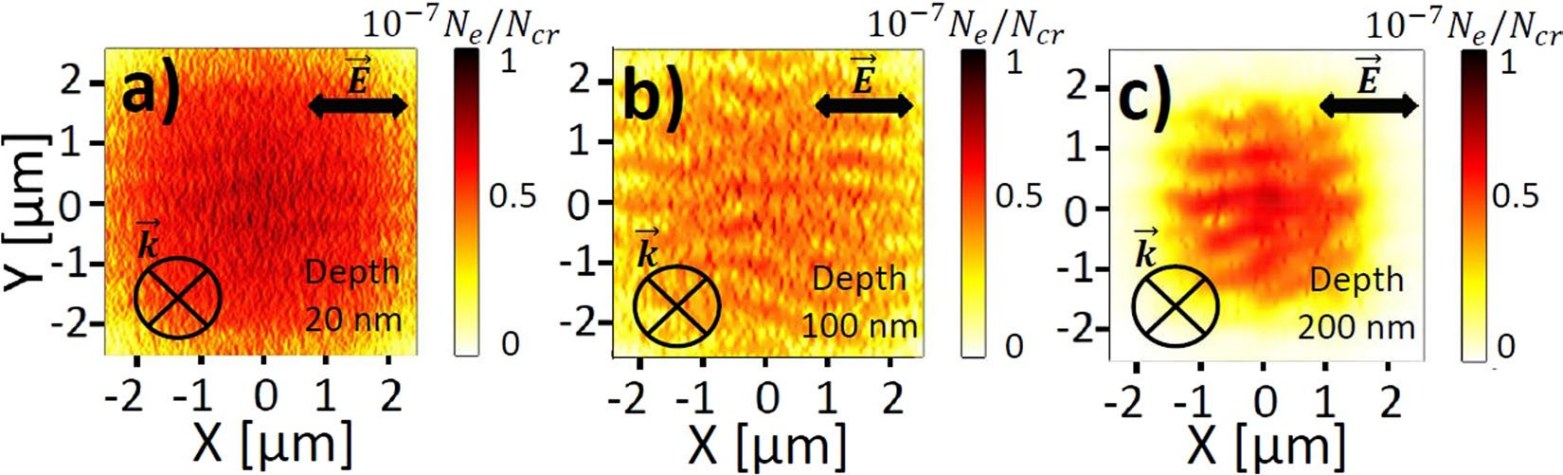
Top-view electron density distributions calculated by 3D nonlinear Maxwell’s equations coupled with electron density equation 40 fs before the pulse peak, corresponding to the interference patterns (**a**–**c**). Pulse duration is 120 fs (FWHM) and laser wavelength *λ* is 800 nm in air. In-plane distributions of the electron density are taken at (**a**) $z=20$ nm, (**b**) $z=100$ nm and (**c**) $z=200$ nm from the silica-air interface. The pulse energy 2 μJ corresponds to a peak fluence of 5 J/cm^2^ with a beam waist ${w}_{0}=5$ μm. Concentration of inhomogeneities on the surface ${C}_{i}=\mathrm{0.1 \% }$. Initial size of inhomogeneities on the surface $r=10$ nm. The electron density is normalized to the critical value ${N}_{cr}=1.74\cdot {10}^{21}$ cm^−3^.

**Figure 4:**
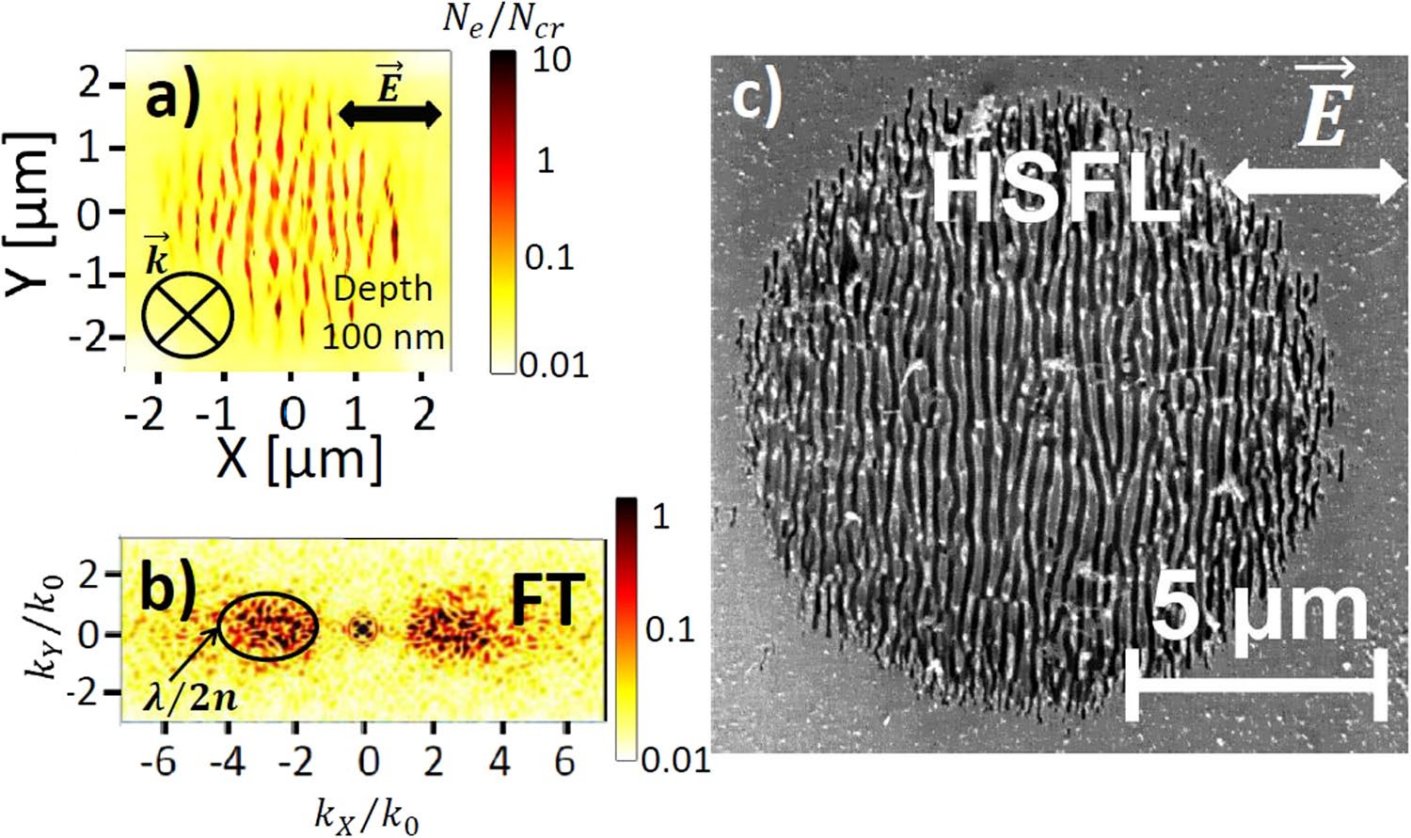
(**a**) Top-view electron density distribution calculated by 3D-FDTD coupled with electron density equation at the pulse peak and (**b**) the corresponding Fourier Transform (FT). Pulse duration is 120 fs (FWHM) and laser wavelength *λ* is 800 nm in air. In-plane distribution of the electron density are taken at $z=100$ nm from the silica-air interface. The pulse energy 2 *μJ* corresponds to a peak fluence of 5 J/cm^2^ with a beam waist ${w}_{0}=5$ μm. Concentration of inhomogeneities on the surface ${C}_{i}=\mathrm{0.1 \% }$. Initial size of inhomogeneities on the surface $r=10$ nm. (**c**) SEM image of the c-SiO_2_ surface after the irradiation with $N=20$ pulses of ${F}_{0}=5.4$ J/cm^2^ peak fluence. Laser wavelength *λ* = 800 nm in air. Pulse duration is 150 fs. The beam waist radius is 18 μm.

**Figure 5:**
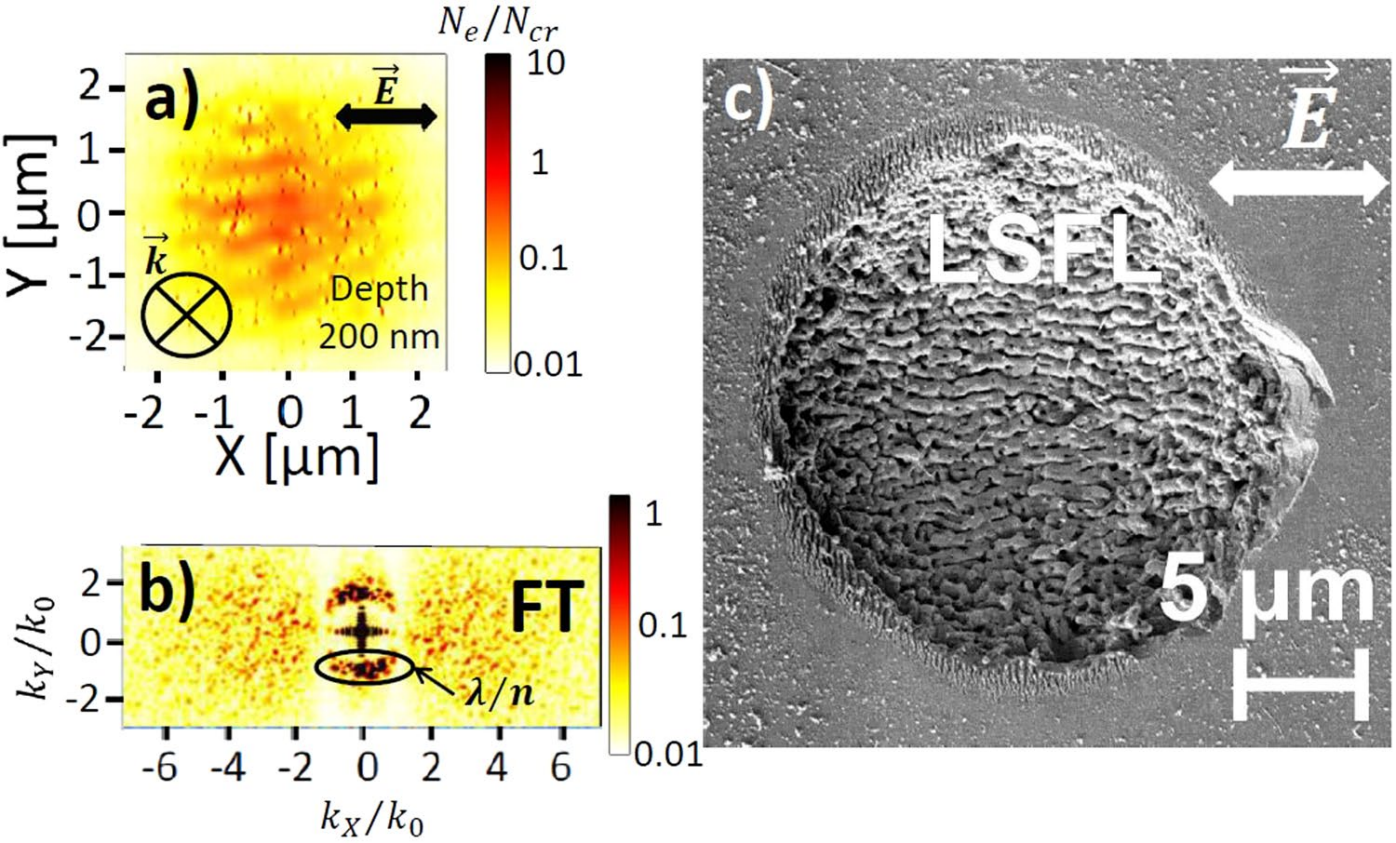
(**a**) Top-view electron density distribution calculated by 3D-FDTD coupled with electron density equation at the pulse peak and (**b**) the corresponding Fourier transform (FT). Pulse duration is 120 fs (FWHM) and laser wavelength *λ* = 800 nm in air. In-plane distribution of the electron density are taken at $z=200$ μnm from the silica-air interface. The pulse energy $2\mu J$ corresponds to a peak fluence of 5 J/cm^2^ with a beam waist ${w}_{0}=5$ μm. Concentration of inhomogeneities on the surface ${C}_{i}=\mathrm{0.1 \% }$. Initial size of inhomogeneities on the surface $r=10$ nm. (**c**) SEM image of a c-SiO_2_ surface after the irradiation with $N=20$ pulses of ${F}_{0}=7.2$ J/cm^2^ peak fluence. Laser wavelength $\lambda =800$ nm in air. Pulse duration is 150 fs. The beam waist radius is 18 μm.

**Figure 6:**
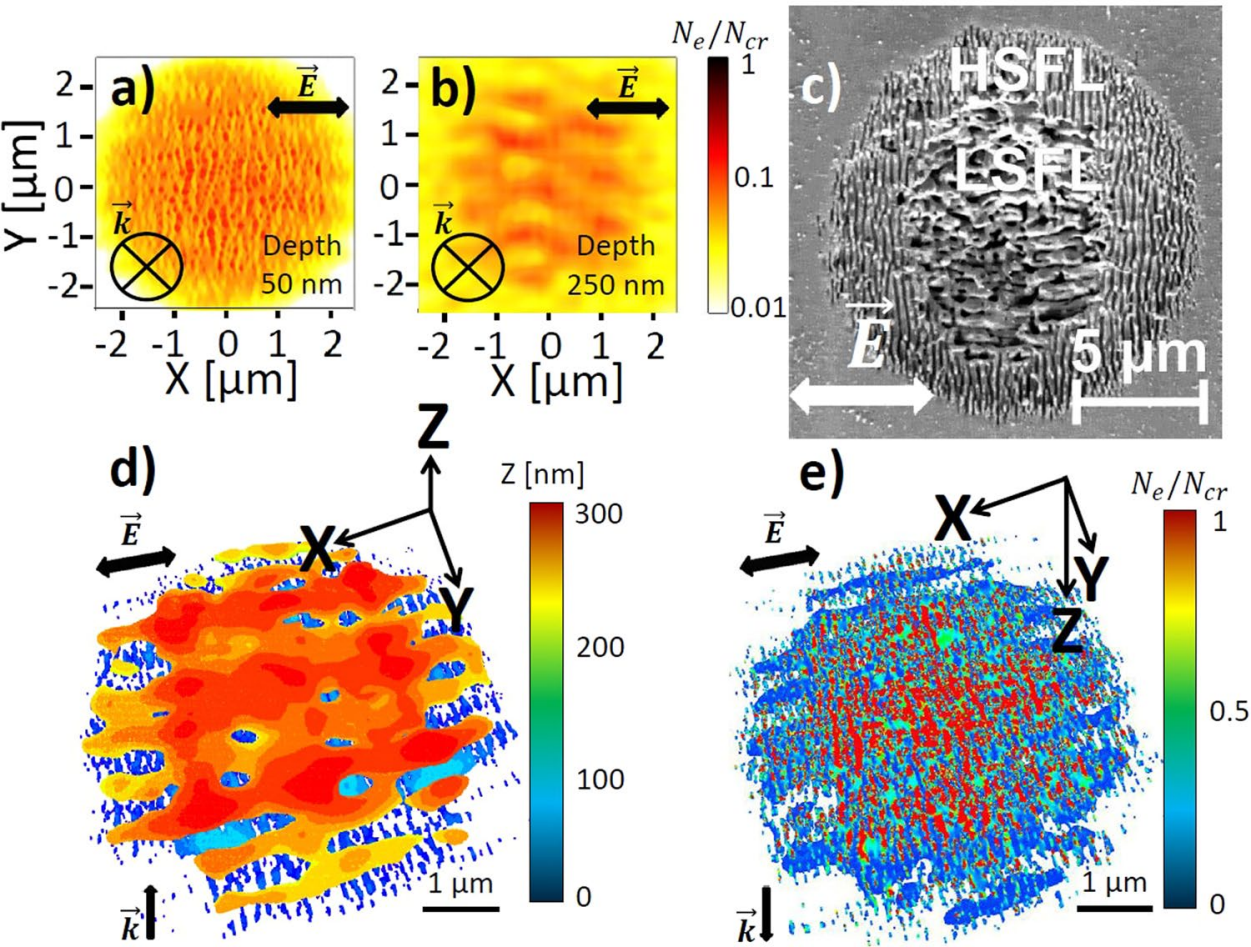
Top-view electron density distributions calculated by 3D-FDTD coupled with electron density equation at the pulse peak with pulse energy $2\mu J$. Pulse duration is 120 fs (FWHM). In-plane distribution of the electron density are taken at (**a**) $z=50$ nm and (**b**) $z=250$ nm from the silica-air interface. The pulse energy 2 μJ corresponds to a fluence of 5 μJ/cm^2^ with a beam waist radius ${w}_{0}=5$ μm. Laser wavelength *λ* is 800 nm in air. Concentration of inhomogeneities on the surface is fixed at ${C}_{i}=\mathrm{0.1 \% }$. Initial size of inhomogeneities on the surface $r=10$ nm. (**c**) SEM image of the c-SiO_2_ surface after the irradiation with $N=20$ pulses of ${F}_{0}=5.8$ J/cm^2^ peak fluency. Laser wavelength $\lambda =800$ nm in air. Pulse duration is 150 fs. The beam waist radius is 18 m. (**d**,**e**) Calculated 3D electron density profiles showing both types of periodic structures (HSFL and LSFL-||) colored as a function of the depth *Z* (**d**) and electron density *N*_*e*_ (**e**). The cutoff for the electron densities is fixed to ${N}_{e} > {10}^{20}$ cm^−3^.

**Figure 7:**
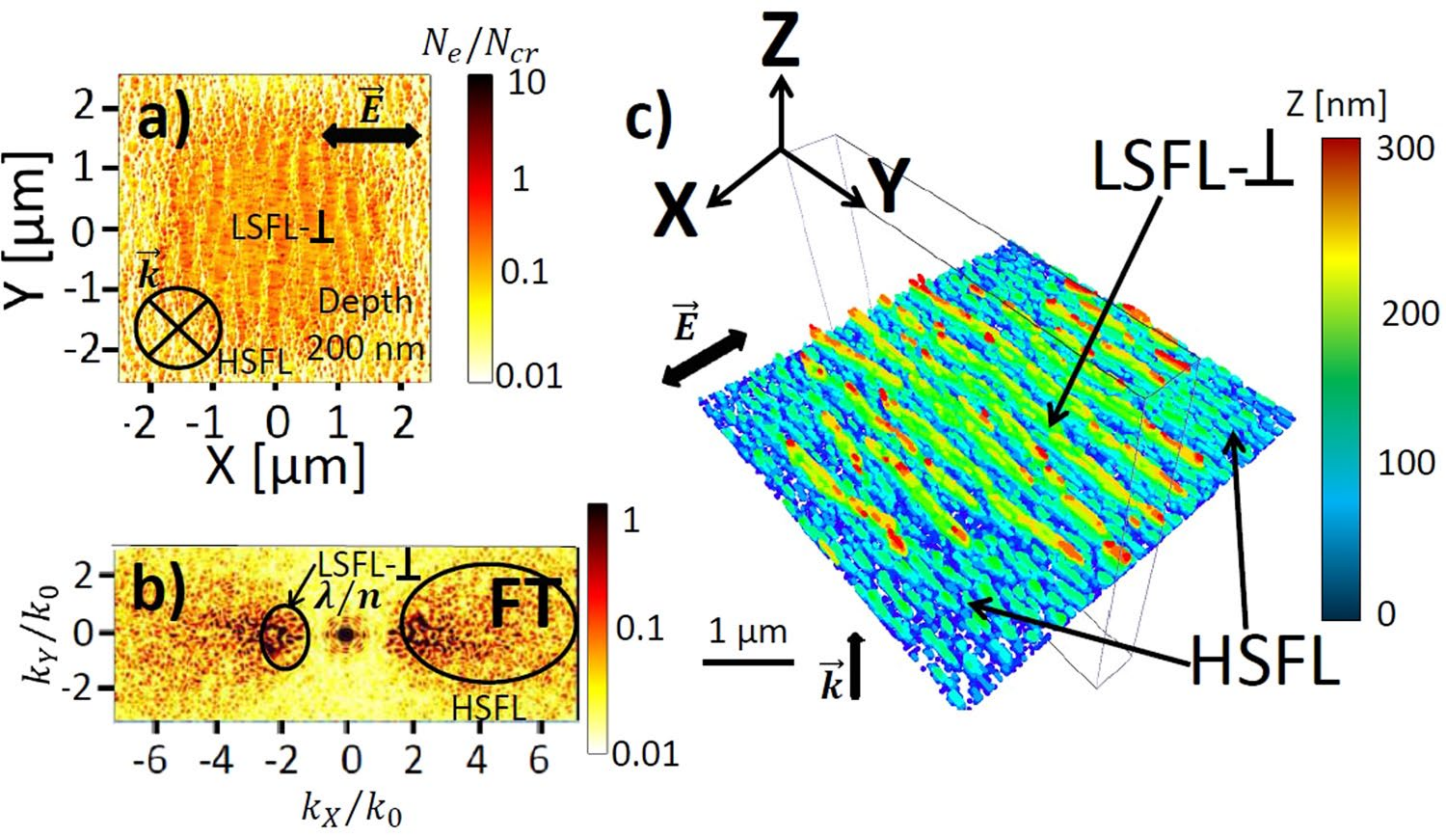
(**a**) Top-view electron density distribution calculated by 3D-FDTD coupled with electron density equation and (**b**) the corresponding Fourier Transform (FT) at the pulse peak with pulse energy 3 μJ corresponding to 7.5 J/cm^2^ with a beam waist radius ${w}_{0}=5$ μm. The snapshot is taken at $z=200$ nm from the silica-air interface. Laser pulse duration is 120 fs (FWHM). (**c**) Corresponding three-dimensional electron density profiles of laser-induced surface modification ${N}_{e} > {10}^{21}$ cm^−3^ colored as a function of depth *Z*. Laser wavelength *λ* is 800 nm in air. The concentration of inhomogeneities on the surface is fixed at ${C}_{i}=\mathrm{0.1 \% }$. Initial size of inhomogeneities on the surface $r=10$ nm.

Figures 3–7 underline the main types of electron density patterns formed by ultrashort pulse irradiation of fused silica at different depths below the surface and for different laser fluences. The calculated electron densities are normalized with the critical carrier density ${N}_{cr}=1.74\cdot {10}^{21}$ cm^−3^ for a wavelength of $\lambda =800$ nm, which is reached when the laser frequency equals the plasma frequency of the laser-generated free electron plasma, leading to resonant absorption of the radiation. At low fluence laser irradiation, the electronic modifications shown in Fig. 3 correspond to the exact intensity maxima, resulted from the interference of the incident laser radiation with the waves scattered from the rough interface. At higher fluences, the local transient changes of the optical properties start playing a decisive role and could trigger specific types of nanostructures, as indicated in Figs 4–7.

Firstly, the electron density distributions far before the temporal pulse peak, corresponding to the negligible change in the optical properties of the dielectric and in the electron densities of the order of ${10}^{-7}{N}_{e}/{N}_{cr}$ and low laser fluence, are investigated. Close to the surface at a depth of 20 nm strongly dispersed subwavelength periodic patterns dominate, oriented perpendicular to $\vec{E}$, see Fig. 3(a). These electron density patterns are the consequences of the interference radiation remnants previously investigated by an electromagnetic approach and referred to as roughness-dependent patterns (or type-r)^19^. It was shown also that the inhomogeneous absorption of optical radiation was triggering the formation of these patterns due to the interference of the incident light with the scattered near-fields of single inhomogeneities^22^. The roughness-dependent features decay rapidly with the depth below the surface^19^, which could be explained by the evanescent nature of the scattered near-fields ${E}_{sca}\propto \mathrm{1/}{a}^{2}$, where *a* is the distance from the single inhomogeneity^50^. We emphasize that the interference between the evanescent scattered near-fields cannot result in the spatial periodic modulation, however, it can contribute to a significant local field enhancement^51^. The spatial periodic modulation is the consequence of the scattered far-field interference with the incident light.

Figure 3(b,c) demonstrate that more pronounced electron density patterns with a characteristic period of the laser wavelength in the dielectric medium and oriented parallel to $\vec{E}$, dominate at the depths $z=100$ nm and $z=200$ nm. These patterns were referred to the dissident interference patterns (or type-d)^19^ and were shown to be triggered by the interference of the incident light with the far-field of inhomogeneities^22^. In fact, their origin, the orientation and the periodicity can be explained as the result of the interference of the incident plane wave with the spherical scattered wave from a single nanosphere in a dielectric medium with the refractive index *n* given by analytical Mie theory^52^. The corresponding interference patterns are shown in Fig. 2(c). The scattered field decays with the distance *a* below the surface as ${E}_{sca}\propto \mathrm{1/}a$ ^50^. We note also that as the electron density of fused silica increases, the real part of the refractive index *n* drops down to the minimum value corresponding to a metallic state with $Re(\varepsilon )={{n}_{0}}^{2}\frac{1-{\omega }^{2}{{\tau }_{e}}^{2}}{1+{\omega }^{2}{\tau }_{e}^{2}} < 0$, where ${n}_{0}=1.45$ is the refractive index of the non-excited silica, $\omega$ is the laser frequency and ${\tau }_{e}$ is the electron collision time (see Supplementary Material for more details). This leads to the larger than $\lambda /{n}_{0}$ periodicity of the interference patterns. In contrast, the imaginary part *k*, causing absorption, increases, therefore, the amplitude of the scattered wave and the interference patterns of type-d decay faster. The parallel oriented structures on the fused silica surface or the LSFL features are commonly observed in experiments for larger pulse energies in the ablated crater^1,33,34^. The calculation results show that the periodic formation of these patterns does not require high electron densities, therefore, non-plasmonic electromagnetic scenario is appropriate to explain the orientation and the periodicity of this kind of ripples^25,53^. Additionally, previous pump-probe diffraction experiments showed that the LSFL structures were seeded in the transparency regime of the dielectric material^54^.

Stronger laser excitation leads to higher electron densities resulting in a significant change of the optical properties of fused silica. High electron density gradients are established between the inhomogeneous random hot spots, resulted from the roughness-dependent patterns, and the surface area affected by multiphoton excitation processes. Although these hot-spots have local metallic properties, we emphasize that the average electron densities in the laser-induced area remain sub-critical. The random inhomogeneities play the role of the seeds for nanoplasma growth and are the reason for the appearance of new periodic perpendicular patterns with subwavelength periodicity at greater depth $z=100$ nm in Fig. 4(a) related to the coherent superposition of the inhomogeneity scattered waves as shown in Fig. 2(b). These nanoplanes continue to grow deeper in the solid, driven by local field enhancement and multiphoton ionization processes. The Fourier transform (FT) of the electron density snapshot shown in Fig. 4(b) reveals a periodicity close to $\lambda /\mathrm{(2}n)\approx 275$ nm and an orientation perpendicular to $\vec{E}$. We note, that the periodicity of the final structures decreases with the increasing inhomogeneity concentration on the rough surface, therefore, even smaller subwavelength periodicities are predicted by the numerical model in the case of higher roughness (not shown here) similar to the case of bulk nanostructuring^10,23^. A similar transition from the random pre-distributed cracks to periodic nanostructures was revealed experimentally on a shot-to-shot basis during ultrashort laser irradiation of fused silica surface^14^. Deep subwavelength HSFL structures oriented perpendicular to laser polarization are commonly observed in dielectrics^1^. The SEM image in Fig. 4(c) shows the nanoripples with a typical $\lambda \mathrm{/(2}n)$ spacing on the surface of fused silica after the irradiation by $N=20$ ultrashort laser pulses.

At a depth of $z=200$ nm, the LSFL-|| structures are clearly seen in Fig. 5(a). These electron-density patterns are likely to be reinforced by the presence of the formed deep HSFL structures^55^. The FT indicates the periodicity close to $\lambda /n\approx 550$ nm and an orientation parallel to $\vec{E}$. The SEM image in Fig. 5c shows the ripples oriented parallel to the laser polarization on the fused silica surface for the fluence higher than the one used to obtain the HSFL structures after $N=20$ pulses irradiation. The experimental results clearly demonstrate that the LSFL structures are observed in the ablation crater, therefore, the material is removed for the lower depths from the surface. Additionally, the position of the LSFL structures deeper than the HSFL structures was reported by two independent experimental groups^33,34^.

To analyze the observed depth-dependent transition from HSFL to LSFL-|| structures, Fig. 6(d,e) show the corresponding three-dimensional electron density modifications. Note that the propagation direction $\vec{k}$ of the laser beam is reversed in Fig. 6(e) to better observe both types of structures. The competition between the structures of two types leads to the formation of a grating-like structure, reported by several independent experimental groups^33,34^. The deposited energy in this case is enough to remove the intensity-enhanced regions of fused silica and to generate the final morphology of the LSFL-|| structures in the center of the laser irradiated zone. Nanostructures obtained by applying the fluence higher than the one for the HSFL but lower than for the LSFL complete morphologies are revealed on SEM image shown in Fig. 6(c). The central laser-irradiated zone is covered with parallel oriented LSFL-|| structures with a period close to $\lambda /n$, whereas the perpendicularly oriented HSFL structures surround the central zone. The calculated three-dimensional electron density profile demonstrated in Fig. 6(e) and the multiphysical study detailed below explain the formation mechanism of the observed ripples morphology. In fact, the electron densities close to the critical value are attained in the center of the laser-irradiated area marked by red color in Fig. 6(e). Corresponding lattice temperatures as high as ${T}_{i}\approx 3500$ K are reached, leading to the efficient material removal. This way, only the morphology created by the interference of the scattered far-field with the incident light and characterized by lower excitation ${N}_{e}\approx 0.5{N}_{cr}$ is conserved in the form of the LSFL-|| structures in the central zone, as well as the surrounding HSFL structures, where the local fluence of the Gaussian beam profile has dropped.

At even stronger excitation, the central laser-induced area turns metallic with $Re(\varepsilon ) < 0$ and perpendicular oriented electron density patterns with a larger periodicity approaching to the laser wavelength are formed at the depths of $z=200$ nm in Fig. 7(a). Their formation is the consequence of the interference of the incident field with the scattered far-field from the rough metallic surface (see Fig. 2d) and was also predicted by analytical Sipe theory^36^ and by linear electromagnetic approach^19^. Our simulations based on the self-consistent model allow us to explain the lacking particularities of mixed LIPSS morphology. Apart from the LSFL-⊥ patterns, the HSFL structures are formed around the quasi-metallic area in Fig. 7(a). To emphasize the same orientation but different periodicity of the electron density patterns, we demonstrate also the corresponding three-dimensional electronic modification in Fig. 7(c). Interestingly, very similar ripples morphologies with the LSFL-⊥ structures in the ablation crater and subwavelength HSFL structures around were observed on the surface of several dielectrics and semiconductors^8,13,39,40^.

### Multipulse feedback

We have shown numerically that a single ultrashort pulse irradiation with initially presented randomly distributed inhomogeneities on the surface or in the bulk of fused silica^23^ leads to the formation of three-dimensional periodic nanoplasmas oriented perpendicular to the laser polarization. However, several pulses are required to form volume nanogratings or surface nanoripples^2,14^. Furthermore, the nanoplanes were shown to consist of nanopores $r=10-20$ nm or less dense matter^15,43^, which means that a certain threshold for nanovoid formation is overcome during ultrashort multipulse laser irradiation and the next pulse interacts with already generated nanovoids. Cavitation below the surface was shown to play a key role in the formation of the inhomogeneously distributed nanovoids and the pre-distributed ripples^56,57^. The laser-induced nanopore formation, preceding the nanogratings development inside the bulk, was also reported^13,15,31^. Therefore, in dielectrics the mechanisms of permanent modification and the multipulse accumulation processes might be as well similar in the case of surface and bulk ultrashort laser processing.

In order to take into account multipulse feedback during ultrashort laser irradiation, the regions where the electron density overcomes the critical value ${N}_{cr}$ are considered to transform into voids with the corresponding permittivity $\varepsilon =1$ and electron density ${N}_{e}=0$. The proposed conditions are close to the void formation thresholds^58^ and the threshold of ${N}_{cr}$ serves as a good approximation and simplification of the real conditions. As in a single pulse irradiation, randomly distributed laser-induced inhomogeneities ($r=10$ nm) with a reduced bandgap ${E}_{g}=5.2$ eV^59^ lower than the electron bandgap of the pristine silica ${E}_{g}=9$ eV^60^ are localized in the volume of fused silica. Keldysh ionization rate ${w}_{pi}$, as well as the photoionization depletion ${\vec{J}}_{pi}$ inside the nanoscale inhomogeneities are recalculated for this particular bandgap (see the Supplementary Material or ref.^49^ for details), giving higher values of the electron density ${N}_{e}$ during pulse duration and, therefore, influencing the near-field enhancement in the vicinity of the inhomogeneities. These hot spots turn into nanovoids up to the beginning of the next pulse. The effective number of pulses *N* is introduced.

**Figure 8:**
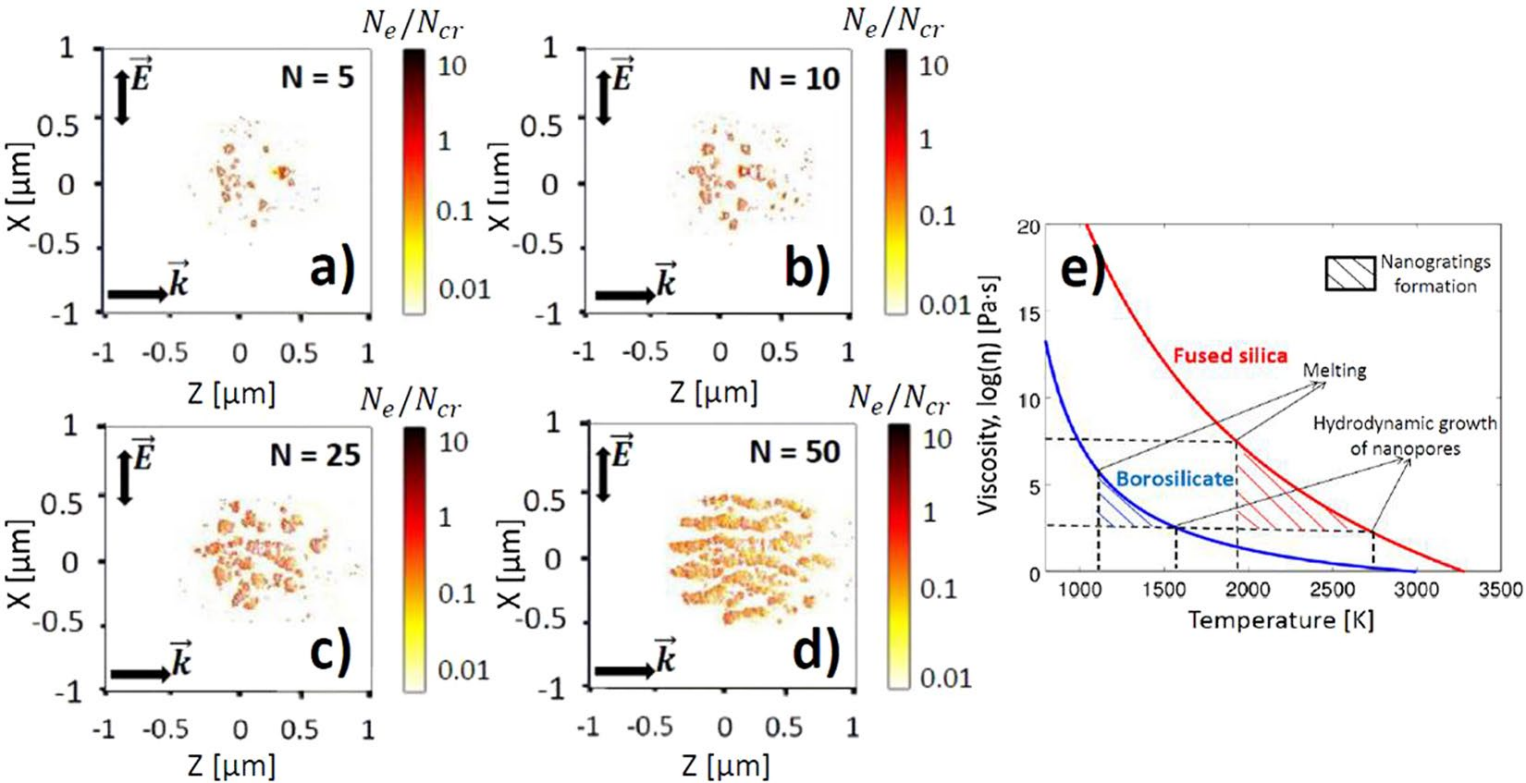
Side-view electron density distributions in the bulk of fused silica at the pulse peak for different effective numbers of pulses (**a**) $N=5$, (**b**) $N=10$, (**c**) $N=25$, (**d**) $N=50$. Pulse duration is 80 fs (FWHM). The pulse energy is fixed to be 200 nJ. The beam waist radius is 1.5 μm and laser wavelength *λ* is 800 nm in air. The initial concentration of randomly distributed inhomogeneities is fixed to ${C}_{i}=\mathrm{0.1 \% }$. The initial size of inhomogeneities $r=10$ nm. (**e**) Temperature thresholds for nanogratings survival in fused silica and borosilicate glass.

Figure 8 shows the electron density snapshots in the bulk of fused silica, taken at different number of pulses and the evolution of nanoplasmas seeded by nanovoids. During the first pulses $(N\le \mathrm{10)}$, there is no periodic organization, the electron density profile is presented by randomly distributed nanovoids surrounded by high density electron plasma as shown in Fig. 8(a,b). Similar non-organized laser-induced modifications were reported in several independent works^15,44,61–63^. At a higher number of pulses, strong local enhancement around both plasma and void-like inhomogeneities contributes to the nanoplasmas elongation in the direction perpendicular to the laser polarization and along the laser beam propagation as evidenced in Fig. 8(c). These void-like inhomogeneities selectively arrange in nanoplanes. The distribution of the nanoplanes after $N=50$ effective pulses is quasi-periodical with subwavelength periodicity close to $\lambda \mathrm{/(2}n)$ in Fig. 8(d) because of the interference of the incident light with the waves scattered from the growing nanovoids. Nanostructures of periodicity smaller than $\lambda \mathrm{/2}n$ can be obtained by considering the initial profile of the nanoscale inhomogeneities with higher concentration, as it was previously discussed in Ref.^23^. Although the collective thermo-mechanical effects are not considered in the multipulse electromagnetic model and require the hydrodynamic approach including the equation of state for fused silica, our simulations clearly demonstrate the electromagnetic origin of periodic formation of nanoporous layers on the shot-to-shot basis.

Another important conclusion is that the discussed electromagnetic multipulse feedback results in the periodic formation of nanogratings, oriented always perpendicular to laser polarization. This way, volume nanogratings with parallel orientation to laser polarization and reported in few semiconductors^43,64^, should have different formation mechanism, either related to different electromagnetic interaction, for instance, coherent superposition of the scattered far-fields from single intrinsic nanovoids, or different multipulse feedback^65^.

The multipulse electromagnetic approach without taking into account the material displacement is valid only if the nanovoids do not grow considerably by hydrodynamic expansion from one pulse to another. In fact, the hydrodynamic expansion intends the void growth in all the stress-induced directions, where the strong temperature gradients are established. In this way, the periodic nanostructures can be erased during the material removal after ultrashort laser irradiation. The condition for viscous growth of a nanovoid can be derived from Rayleigh-Plesset equation ${P}_{e}={\rm{\Delta }}P{\tau }_{cool}/\eta \gg 1$, where ${P}_{e}$ is Peclet number, ${\rm{\Delta }}P\approx -\frac{3}{2}{N}_{a}{k}_{B}{T}_{i}$ is the pressure induced by tensile stress within the formed nanovoid, ${N}_{a}$ is the atomic density of glass, $\eta ({T}_{i})$ is the temperature-dependent viscosity, and ${\tau }_{cool}=\rho {C}_{i}{w}_{0}^{2}/{\kappa }_{i}$ is the cooling time^42,66^. The Peclet number here defines the ratio between the viscous deformation and diffusion rates. The higher this number is, the stronger nanovoid growth is expected and the less important is the role of the lattice cooling. Figure 8(e) shows the temperature dependencies of viscosities for fused silica and borosilicate glasses taken from Ref.^67^. The melting temperature and the temperature, satisfying the condition for viscous growth of the navoids ${P}_{e}=1$ serve as the local indicators for nanogratings formation and erasure. One can note, that the laser parameter window for borosilicate glass is significantly narrower than for fused silica since borosilicate glass has a lower viscosity at the same temperatures and a larger thermal expansion coefficient and laser-induced stresses. This results in larger Peclet number ${P}_{e}$. Therefore, it is more difficult to find suitable laser regimes for inscribing periodic structures inside borosilicate glasses^31,68^. Apart from the thermo-mechanical mechanisms resulting in the formation of nanovoids, another multipulse feedback mechanisms may play the role of the precursors for the inhomogeneities in glasses during first pulses irradiation, such as formation of electronic defect states^54^ or chemical changes^69^, both related to the lowering of the band gap in glasses.

### Dynamics of nanovoid formation and material removal

**Figure 9:**
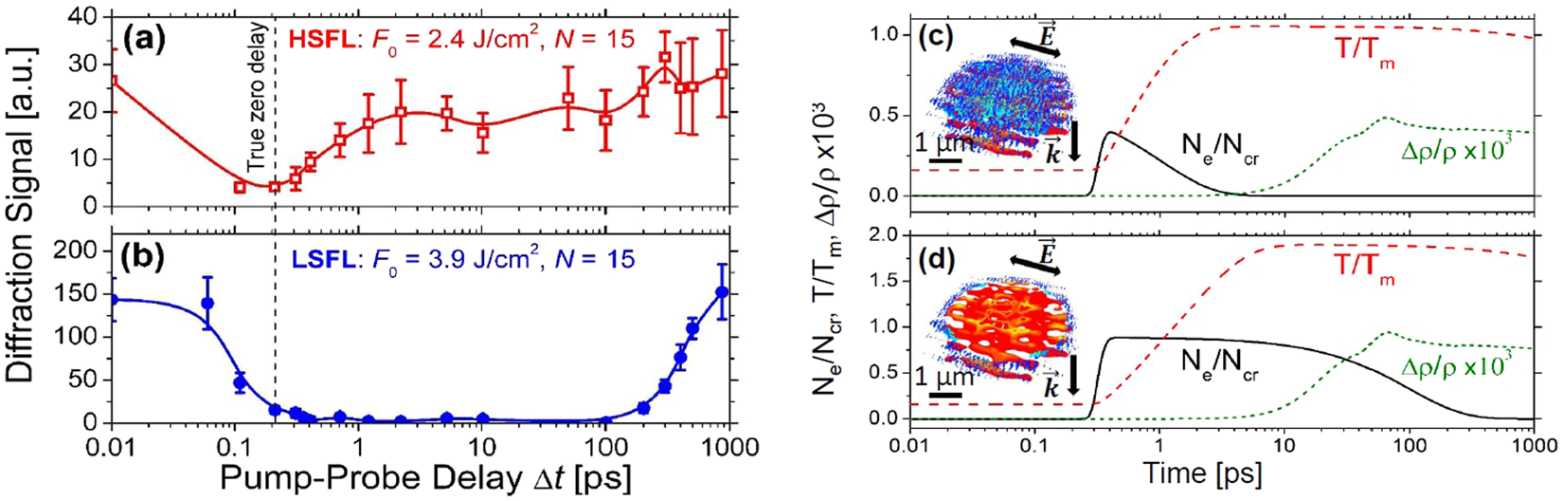
(**a**,**b**) Trans-illumination diffraction signals as a function of the time-delay $${\rm{\Delta }}t$$ measured upon fused silica/air surface irradiation by $$N=15$$ pulses (**a**) with a peak fluence $${F}_{0}=2.4$$ J/cm^2^ (the regime where the HSFL structures were observed) and (**b**) with a peak fluence $${F}_{0}=3.9$$ J/cm^2^ (the regime where the LSFL structures were observed). Curve in (**b**) is taken from ref.^54^. Pulse duration is 50 fs (FWHM). Laser wavelength *λ* is 800 nm in air. The beam waist radius is 46 μm. Note that for the semi-logarithmic representation, all delay values $${\rm{\Delta }}t$$ were shifted artificially by 0.2 ps. (**c**,**d**) Time evolution of calculated maximum electron density, lattice temperature and density for the peak fluences (**c**) $${F}_{0}=2.4$$ J/cm^2^ and (**d**) $${F}_{0}=3.9$$ J/cm^2^ over the laser-induced area. Concentration of inhomogeneities on the surface is fixed at $${C}_{i}=\mathrm{0.1 \% }$$. The cut-offs of the 3D electron density profiles are set to be (**c**) $${N}_{e} > {10}^{20}$$ cm^3^ and (**d**) $${10}^{20}$$ cm^−3^ $$ < {N}_{e} < {10}^{21}$$ cm^−3^.

To get a deeper insight in glass decomposition processes taking place during and after ultrashort laser interaction and the dynamics of HSFL and LSFL, the time-resolved experimental measurements of diffraction from the surface as well as the numerical analysis of time-dependent evolution of the modification are performed. The experimental trans-illumination set-up is described elsewhere^54^. A summary is provided in the Methods section at the end of the manuscript. In brief, a pump-probe diffraction experiment with a temporal resolution <0.1 ps was performed using the fundamental (800 nm) radiation for generating LIPSS at the sample surface (*N* pulses per spot). The signal of the $(N+\mathrm{1)}$-th frequency-doubled, delayed probe pulse (400 nm) diffracted at the LIPSS was acquired versus the pump-probe delay time ${\rm{\Delta }}t$ by a photodetector. The recordings of the first order femtosecond time resolved diffraction from 0.01 ps to 1 ns are shown in Fig. 9(a,b). The diffraction signals from the HSFL and LSFL structures modification indicating the presence of the periodic structures are measured on fused silica surface after $N=15$ pulses irradiation. The decay of the diffraction signal results from screening of the diffracted probe beam by the electron plasma, which covers the void-like structure formed by previous laser pulses^54^. Thus, the decay of the electron plasma corresponding to the lower fluence ${F}_{0}=2.4$ J/cm^2^ can be related to the electron plasma recombination ${\tau }_{rec}\approx 1$ ps and for the higher fluence ${F}_{0}=3.9$ J/cm^2^ the value is close to ${\tau }_{rec}=100$ ps. In fact, the recombination time was shown to increase with the increasing electron densities/temperatures^58,70^. The experimental measurements are consistent with the time-resolved free carrier density evolution on the surface^70^ and inside fused silica bulk^58^.

To estimate the corresponding electron densities, lattice temperatures and density changes, a multiphysical model is applied, detailed in the Supplementary Material. This model consists of nonlinear Maxwell’s equations coupled with a rate equation, as in the calculations above, and is additionally supplemented by electron-ion temperature and thermo-elastoplastic models. The time evolution of the maximum electron densities (solid black curves) and lattice temperatures (dashed red curves) corresponding to the fluences (c) ${F}_{0}=2.4$ J/cm^2^ and (d) ${F}_{0}=3.9$ J/cm^2^ and to electron recombination times (c) ${\tau }_{rec}=1$ ps and (d) ${\tau }_{rec}=100$ ps are shown in Fig. 9(c,d). The electron densities are lower and near the critical value ${N}_{cr}$ for two corresponding fluences. The maximum temperature for the lower fluence are of the order of ${T}_{melt}$, whereas it is significantly higher in the second case of the order of $T\approx 3500$ K. This value is higher than the boiling point for fused silica ${T}_{boil}=2500-3200$ K^71–73^, and corresponds also to high ${P}_{e} > 1$ numbers. These results indicate that the material is removed for the higher fluence, the HSFL structures are erased in the center of the heat-affected zone. Only the LSFL-|| structures which are generated by far-field interaction deeper in the material remain in the ablated crater. This way, we explain the dominance of LSFL-|| over HSFL structures for high fluences. We underline that the transition from HSFL to LSFL-|| as dominant surface morphologies is only due to the removal of the material but not due to the metallic properties of the material, as the electron densities are below in Fig. 9(c) or near the critical value in Fig. 9(d). The numerical calculations also indicate that LSFL-⊥ structures can not be easily formed in fused silica, because the material will be efficiently removed by the ablation processes if metallic properties of glass ${N}_{e} > ({\varepsilon }_{\infty }+\mathrm{1)}\frac{{\omega }^{2}{\tau }_{e}^{2}+1}{{\omega }^{2}{\tau }_{e}^{2}}\cdot {N}_{cr}/2$ are attained during the ultrashort pulse irradiation. This statement is directly supported by the SEM image of LSFL-|| shown in Fig. 5(c). These structures were generated at a peak fluence of 7.2J/cm^2^ in the bottom of a several micrometer deep crater, while the carrier densities calculated in the near surface region are similar to those shown in Fig. 7, i.e. LSFL-⊥ patterns. Note that the latter structures on fused silica were only reported for extremely short pulse durations (5 fs) and at ${F}_{0}\approx 6.9$ J/cm^2^ ^37^, featuring electron densities in the metallic regime $({N}_{e} > {N}_{cr})$.

The corresponding temporal evolution of the density changes due to the material expansion and rarefaction ${\rm{\Delta }}\rho /\rho  < 0$ are also shown as dotted green curves in Fig. 9(c,d). The maximum density change is attained when the pressure wave is launched in fused silica, corresponding to time of $\delta T\approx 65$ ps for both irradiation fluences. The corresponding strain rate is estimated as $\varsigma =-{\rm{\Delta }}\rho /(\rho T)$. Then, the Grady’s criterion for spall in liquid^41^ detailed in Supplementary Material is applied to estimate the time required for the void formation. The calculated temperature profiles shown in Fig. 9(c,d) are considered to take into account the rapid cooling of the lattice after ultrashort laser excitation. The viscosity is evaluated according to the temperature dependent curve illustrated in Fig. 8(e) for fused silica. The model predicts that the nanocavitation in $r=10$ nm fragments takes place at $t=\sqrt{6\sigma /BR{\varsigma }^{2}}\approx 980$ ps where the local electron densities ${N}_{e}\approx 1.5\cdot {10}^{21}$ cm^−3^ are attained, i. e. for ${N}_{e}$ close to the critical value ${N}_{cr}$. For the lower fluence and the electron densities not exceeding ${N}_{e} < 7\cdot {10}^{20}$ cm^−3^ the lattice cools down faster than the void formation takes place. Additionally, the threshold parameters for nanopore formation by ultrashort laser are estimated by applying Grady’s criterion for spall in liquid: lattice temperatures slightly exceeding the melting temperature $T\approx 2050$ K and the laser-induced strain rates of order $\varsigma \approx {10}^{7}$s^−1^.

## Conclusions

The following conclusions can be drawn from the performed numerical and experimental study. Volume nanogratings and subwavelength surface nanoripples (HSFL) have similar formation mechanisms, and the presence of initial inhomogeneities, electronic defects or scattering centers is required to start the nanoplasma growth. The orientation of the nanoplasmas is defined by the local field enhancement perpendicular to the laser polarization, whereas the subwavelength periodicity is the consequence of the coherent superposition of scattered waves emitted by nanoplasmas. The process of the nanostructure formation does not require that fused silica glass turns into a metallic state, because the significant local field enhancement is achieved at the tips even of a void nanoplane providing the growth on a shot-to-shot basis.

The HSFL nanoripples with the orientation perpendicular to the laser polarization grow driven by the interference between the incident field and the scattered near-field below the surface. In contrast, the LSFL classical ripples with the orientation parallel or perpendicular to the laser polarization are the results of the interference of the incident light with the far-field of rough non-metallic or metallic surfaces. Therefore, they are formed dominantly on greater depths and at higher laser fluences or irradiation dose. The numerical results indicate non-metallic nature of the transition between HSFL and LSFL-|| and the metallic nature of the LSFL-⊥ structures. Based on the time-resolved diffraction measurements, as well as the developed electromagnetic and thermo-mechanical models, the mixed ripple morphologies are explained. The HSFL structure erasure is predicted in the central laser-irradiated area, where the material is efficiently removed and only the deeper far-field induced LSFL structures remain. The results are supported by series of experimental SEM images elucidating the polarization-dependent ripple morphology, observed on the surface of fused silica.

Regarding the bulk nanoprocessing, the path to the periodic nanostructure formation might require higher number of pulses to create firstly the sufficient bulk nanoroughness in the form of the laser-induced nanopores, e. g. after STE or color center formation upon multi-pulse irradiation. Furthermore, volume nanogratings are likely to be formed only in transparent materials with a high viscosity and a low thermal expansion coefficient, enabling formation of nanopores. These nanopores act as scattering centers embedded in the bulk and seeds for nanoplasma growth perpendicular to the laser polarization. The laser parameter window for the nanostructure formation is limited by high temperatures, at which the nanopores rapidly grow via hydrodynamic expansion. According to our estimations, the narrower window is predicted for borosilicate than for fused silica glass, which agrees well with the experimental observations.

## Methods

### Computational method

The full details of the model equations and the parameters used in the calculations are given in the Supplementary Material. The numerical model used to obtain the results in Figs 2–7 is based on the three-dimensional nonlinear Maxwell’s equations coupled with rate equation

1 $$\{\begin{array}{ccc}\frac{{\rm{\partial }}\overrightarrow{E}}{{\rm{\partial }}t} & = & \frac{\bigtriangledown \times \overrightarrow{H}}{{\varepsilon }_{0}{\varepsilon }_{{\rm{\infty }}}}-\frac{1}{{\varepsilon }_{0}{\varepsilon }_{{\rm{\infty }}}}({\overrightarrow{J}}_{p}+{\overrightarrow{J}}_{pi})\\ \frac{{\rm{\partial }}\overrightarrow{H}}{{\rm{\partial }}t} & = & -\frac{\bigtriangledown \times \overrightarrow{E}}{{\mu }_{0}}\\ \frac{{\rm{\partial }}{\overrightarrow{J}}_{p}}{{\rm{\partial }}t} & = & \frac{{\overrightarrow{J}}_{p}}{{\tau }_{e}}+\frac{{e}^{2}{N}_{e}}{{m}_{e}}\overrightarrow{E}\\ \frac{{\rm{\partial }}{N}_{e}}{{\rm{\partial }}t} & = & \frac{{N}_{a}-{N}_{e}}{{N}_{a}}{w}_{pi}+{W}_{av}-\frac{{N}_{e}}{{\tau }_{rec}}\end{array},$$

where $\vec{E}$ is the electric field, $\vec{H}$ is the magnetizing field, ${\varepsilon }_{0}$ is the free space permittivity, ${\mu }_{0}$ is the free space permeability, ${\vec{J}}_{p}$ and ${\vec{J}}_{pi}$ are the polarization and the photoionization currents, ${N}_{e}$ is the time-dependent electron density, ${\tau }_{e}$ is the electron collision time, ${m}_{e}$ is the electron mass, ${\tau }_{rec}$ is the electron recombination time, ${N}_{a}$ is the saturation density, ${\varepsilon }_{\infty }=2.105$ is the permittivity of the non-excited fused silica. The details of the numerical method are described in ref.^49^. The electrons in the conduction band are generated by Keldysh photoionization ${w}_{pi}$ and avalanche ionization ${W}_{av}$ mechanisms, detailed in the Supplementary Material. The properties of the glass are modified via heating described by a Drude model. The initial source is introduced as a linear polarized Gaussian focused beam^10^.

Then, two-dimensional calculations are performed to evaluate the temperature evolution and the density evolution depending on the laser fluence. This approach consists of nonlinear Maxwell’s equations, rate equation, electron-ion temperature model, thermo-elastoplastic wave equations and the Euler’s equation for the material density. The model is complemented by Grady’s criterion for spall in liquid^41^ and Rayleigh-Plesset hydrodynamic equations^42^ to describe the void formation and growth after ultrashort laser irradiation. The equations are detailed and the thermo-mechanical properties for fused silica and borosilicate glass are given in the Supplementary Material.

### Experimental method

For “static” *ex-situ* fs-laser irradiation experiments, high-purity double-side polished single-crystalline synthetic quartz samples [c-SiO_2_,(0001) crystal cut orientation] were purchased from CrysTec GmbH, Berlin, Germany. A commercial Ti:sapphire fs-laser system (TRA-1000, Clark-MXR) was used to generate linearly polarized laser pulses of 150 fs duration at 800 nm central wavelength at a pulse repetition frequency of 150 Hz. Multiple (*N*) pulses were selected by an electro-mechanical shutter and were focused by a spherical lens ($f=100$ mm) under normal incidence onto the sample surface (Gaussian beam $\mathrm{1/}{e}^{2}$-radius ${w}_{0}\approx 18$ μm). The peak fluences ${F}_{0}$ of the Gaussian-like beam profile in front of the sample were determined according to a method proposed by Liu^74^. The uncertainty of the laser fluences is less than 20%. All irradiations were performed in air environment. Similar static irradiation experiments were performed also on fused silica samples (Suprasil, Heraeus GmbH). Although the damage threshold of fused silica is lower than that of quartz by $\approx \mathrm{10 \% }$ for pulse numbers less than ten per spot, we have not observed significant differences between both types of silica samples with respect to the morphology of the LIPSS^33^.

“Dynamic” *in-situ* trans-illumination pump-probe experiments were performed to reveal the temporal built-up of the optical diffraction at LSFL and HSFL generated under suitable multi-pulse irradiation conditions. The experimental setup is described in detail in ref.^54^. In brief, linearly polarized laser pulses with a duration of $\approx 50$ fs and a repetition rate of 250 Hz were provided by a Ti:sapphire fs-laser system (Spitfire, Spectra Physics). The pump-beam at the fundamental wavelength (${\lambda }_{Pump}=800$ nm) was focused in air onto the front surface of a 10 × 10 × 1 mm^3^ double-side polished fused silica plate (Suprasil, Heraeus GmbH) using a $f=200$ mm lens under normal incidence to generate regular LIPSS over the central irradiated area. The corresponding Gaussian beam radius was ${w}_{\mathrm{0,}Pump}\mathrm{(1/}{e}^{2})\approx 46$ μm. For a fixed number of $N=15$ pump pulses and at a suitable peak fluence either LSFL-|| (${F}_{0}=3.9$ J/cm^2^) with periods $550-700$ nm, or HSFL (${F}_{0}=2.4$ J/cm^2^) with periods between 200 and 300 nm were “prepared” on a non-irradiated site of the silica surface before each pump-probe irradiation. For that, a small fraction of the original fs-laser beam was separated as a probe-beam, frequency-doubled in barium borate crystal (${\lambda }_{Probe}=400$ nm), delayed with respect to the pump pulse by an optical delay-line consisting of a retroreflector and a motorized linear translation stage, and focused by 60 mm achromatic lens under an angle of $\alpha \approx {15}^{\circ }$ to the surface normal into the center of the pump spot ${w}_{\mathrm{0,}Probe}\mathrm{(1/}{e}^{2})\approx 10$ μm. The beam resulting from ${1}^{st}$-order diffraction of a single p-polarized probe-pulse on the grating-like LSFL during the ($N+1$) pump-probe irradiation was collected in transmission geometry (i.e. through the silica sample) with a $f=25$ mm lens and focused onto a silicon photodiode (DET-10 A/M, Thorlabs), which was coupled to an oscilloscope. Residual 800 nm radiation was eliminated by an infrared-cutting bandpass filter (Schott, BG 39) placed in front of the photodiode. All measurements were conducted ten times, providing the mean value and the standard deviation of the diffraction signal peak intensity. For probing the dynamics of the HSFL formation, due to their smaller periods, the setup was modified to allow diffraction. In that case, the angle of incidence of the probe-beam was chosen at $\alpha \approx {45}^{\circ }$ and the detection path (${1}^{st}$-order diffraction) was re-arranged accordingly. The temporal resolution of the pump-probe setup was better than 0.1 ps.

## Electronic supplementary material

Supplementary Info
