# Supplementary material for: Spontaneous periodic ordering on the surface and in the bulk of dielectrics irradiated by ultrafast laser: a shared electromagnetic origin
Source: Sci Rep. 2017 Sep 26;7:12306. doi: 10.1038/s41598-017-12502-4 (PMC5615049; doi:10.1038/s41598-017-12502-4)
Supplement: Supplementary file 1 — Supplementary Info [file 41598_2017_12502_MOESM1_ESM.pdf]

Supplementary material to "Spontaneous periodic ordering on the surface and in the bulk of dielectrics irradiated by ultrafast laser: a shared electromagnetic origin"

Anton Rudenko and Jean-Philippe Colombier*
Univ Lyon, UJM-St-Etienne, Laboratoire Hubert Curien, CNRS UMR 5516, F-42000, Saint-Etienne, France

Sandra Höhm and Arkadi Rosenfeld
Max-Born-Institut für Nichtlineare Optik und Kurzzeitspektroskopie (MBI), Max-Born-Straβe 2A, D-12489 Berlin, Germany

Jörg Krüger and Jörn Bonse
Bundesanstalt für Materialforschung und -prüfung (BAM), Unter den Eichen 87, D-12205 Berlin, Germany

Tatiana E. Itina†
Univ Lyon, UJM-St-Etienne, Laboratoire Hubert Curien, CNRS UMR 5516, F-42000, Saint-Etienne, France and ITMO University, Kronverskiy pr. 49, St. Petersburg, Russia
(Dated: June 29, 2017)

* jean-philippe.colombier@univ-st-etienne.fr
† tatiana.itina@univ-st-etienne.fr

I. MAXWELL'S EQUATIONS COUPLED WITH RATE EQUATION

The numerical model is based on the three-dimensional nonlinear Maxwell's equations coupled with a rate equation

$$\begin{cases} \frac{\partial \vec{E}}{\partial t} = \frac{\nabla \times \vec{H}}{\epsilon_0 \epsilon_\infty} - \frac{1}{\epsilon_0 \epsilon_\infty}(\vec{J}_p + \vec{J}_{pi}) \\ \frac{\partial \vec{H}}{\partial t} = -\frac{\nabla \times \vec{E}}{\mu_0} \\ \frac{\partial \vec{J}_p}{\partial t} = -\frac{\vec{J}_p}{\tau_e} + \frac{e^2 N_e}{m_e}\vec{E} \\ \frac{\partial N_e}{\partial t} = \frac{N_a - N_e}{N_a} w_{pi} + W_{av} - \frac{N_e}{\tau_{rec}}, \end{cases} \quad (1)$$

where $\vec{E}$ is the electric field, $\vec{H}$ is the magnetizing field, $\vec{J}_p$ is the polarization current describing the electron heating and $\vec{J}_{pi}$ is the photoionization depletion current taking account the reduction in optical pulse energy required to ionize the medium, N_e is the time-dependent electron density, $\tau_e = 0.5$ fs is the electron collision time [1], m_e is the electron mass, $\tau_{rec} = 1$ ps is the electron recombination time [2], $\epsilon_\infty = 2.105$ is the permittivity of the non-excited fused silica, $N_a = 2 \cdot 10^{22}\text{cm}^{-3}$ is the saturation density [3]. The details of the numerical method are described in Ref. [4]. The electrons in the conduction band are generated by Keldysh photoionization w_{pi} [5] and avalanche ionization W_{av} mechanisms [6]. The properties of the glass are modified via heating described by a Drude model.

A focused linear polarized Gaussian beam source is used to simulate the irradiation by laser as follows

$$E_x(t,x,y,z) = \frac{w_0}{w(z)} \exp\left[-\frac{r^2}{w(z)^2} - ikz - ik\frac{r^2}{2R(z)} + i\varsigma(z) - \frac{(t-t_0)^2}{\theta^2} \right], \quad (2)$$

where w_0 is the beam waist radius, $w(z) = w_0\sqrt{1 + (\frac{z}{z_R})^2}$ is the depth variation of the beam radius at which the field amplitude drops to $1/e$ of their axial values, $z_R = \pi {w_0}^2 n_0/\lambda$ is the Rayleigh length, $r = x^2 + y^2$ is the radial distance from the beam waist, $R(z) = z[1 + (\frac{z_R}{z})^2]$ is the radius of curvature of the wavefront comprising the beam, and $\varsigma(z) = \arctan(\frac{z}{z_R})$ is the Gouy phase shift. The waist beam is taken $w_0 = 5$ µm while investigating the electron density distributions and LIPSS morphologies in Figs. 2-7. The numerical results for multiphysical modeling in Fig. 9(b, d) and for the multipulse simulations in Fig. 8(a, d) are obtained by considering the restricted laser-irradiated zone of $w_0 = 1.5$ µm. The input laser pulse energy is defined as

$$W = \sqrt{\frac{\pi}{2}} \frac{\pi w_0^2}{2} \theta I, \quad (3)$$

where θ is the pulse duration (FWHM), $I = \frac{1}{2}\sqrt{\frac{\epsilon_0 \epsilon_\infty}{\mu_0}} \left|\vec{E}\right|^2$ is the intensity [7, 8], and the peak laser fluence corresponds to $F_0 = W/(\frac{\pi {w_0}^2}{2})$.

The avalanche ionization rate is derived from the Drude model [9–11], where the heating of free electrons is associated with the absorption coefficient. In this case, the avalanche ionization rate is described via the Drude formalism as

$$W_{av} = \frac{e^2 \tau_e N_e \left|\vec{E}\right|^2}{n_0 c \epsilon_0 m_e E_g (1 + \omega^2 {\tau_e}^2)(1 + {m_e}^*/m_e)} \frac{N_a - N_e}{N_a}, \quad (4)$$

where ${m_e}^* = 0.5 m_e$ is the reduced electron mass [12], $E_g = 9$ eV is the electron band gap, and $n_0 = 1.45$ is the refractive index of the non-excited fused silica [11].

The real and the imaginary parts of the dielectric permittivity $\epsilon = \epsilon_1 + i \cdot \epsilon_2$ are derived from a simple Drude model with time-varying electron density N_e as follows

$$\begin{cases} \epsilon_1(N_e) = \epsilon_\infty - \frac{e^2 N_e}{m_e(\omega^2 + {\nu_e}^2)} \\ \epsilon_2(N_e) = \frac{e^2 N_e \nu_e}{m_e \omega(\omega^2 + {\nu_e}^2)}, \end{cases} \quad (5)$$

where $\nu_e = {\tau_e}^{-1}$ is the electron collision frequency. The permittivity of the material is directly related to the optical properties of the medium $\epsilon = (n + ik)^2$, where n is the refractive index, which dictates the phase velocity information,

and k is the extinction coefficient, which dictates the amount of absorption loss while propagating through the material. One can derive the expressions for them as $n = \sqrt{(\sqrt{\epsilon_1^2 + \epsilon_2^2} + \epsilon_1)/2}$ and $k = \sqrt{(\sqrt{\epsilon_1^2 + \epsilon_2^2} - \epsilon_1)/2}$. Finally, the absorption coefficient is related to the extinction coefficient as $\alpha_{abs} = 4\pi k/\lambda$.

As the electron density N_e increases, the real part of the permittivity ϵ_1 decreases and the imaginary part ϵ_2 increases. This way, one can find a minimum value of the attained refractive index $n(N_e)$ by finding the extremum of the function. Thus, the extremum is defined by $N_e = 2{n_0}^2 \frac{\epsilon_0 m_e \omega^2 {\tau_e}^2}{e^2(1+\omega^2\tau_e^2)}$ and the minimum value $n_{min} = n_0\sqrt{1/(1+\omega^2\tau_e^2)}$ corresponds to $\epsilon_1 = {n_0}^2 \frac{1-\omega^2{\tau_e}^2}{1+\omega^2\tau_e^2}$. One can find that for $\tau_e = 0.5$ fs and the laser wavelength $\lambda = 800$ nm, this value $\epsilon_1 < 0$, therefore, the refractive index decreases up to the minimum value, corresponding to metallic state of glass. Note, that the transition to metallic state with $\epsilon_1 < 0$ takes place for $n(N_e) = \sqrt{\epsilon_2/2} = n_0/\sqrt{2\omega\tau_e} > n_{min}$, i. e. for the electron densities lower than the one corresponding to the minimum refractive index. Therefore, the maximum theoretical LSFL periodicity defined by the expression $\lambda/n(N_e)$ is estimated to be $\Lambda_{max} = \lambda\sqrt{2\omega\tau_e}/n_0$.

II. ELECTRON-ION HEAT TRANSFER EQUATIONS

The ionization process locally transforms dielectric material into an absorbing plasma with metallic properties. The electrons in the conduction band are heated by the laser, and transfer their energy to the lattice. Heating of the dielectric can be described by the two-temperature model, and the energy conservation law as follows

$$\begin{cases} C_e\dfrac{\partial T_e}{\partial t} = \nabla\cdot(\kappa_e\nabla T_e) - \gamma_{ei}(T_e - T_i) + I\alpha_{abs} \\ C_i\dfrac{\partial T_i}{\partial t} = \nabla\cdot(\kappa_i\nabla T_i) + \gamma_{ei}(T_e - T_i) - B\beta T_0\dfrac{\partial}{\partial t}(\nabla\cdot\vec{u}), \end{cases} \tag{6}$$

where $\gamma_{ei} = C_e/\tau_e$ is the electron-lattice coupling factor, $C_e = \frac{3}{2}k_B N_e$ and $C_i(T_i)$ are the electron and the lattice heat capacity respectively, $\kappa_e = 2{k_B}^2\mu_e N_e T_e/e$ and $\kappa_i(T_i)$ are the electron and the lattice thermal conductivities, α_{abs} is the bremstrahlung absorption coefficient related to the extinction coefficient (imaginary part of the optical refractive index) as $\alpha = 4\pi k/\lambda$, $\vec{u}$ is the displacement vector, β is the coefficient of thermal expansion, B is the bulk elastic modulus, $\mu_e = 3\cdot 10^{-5}\frac{m^2}{V\cdot s}$ is the electron mobility [13], and $k_B = 1.38\cdot 10^{-23}$ m^{2}kg$\cdot$s^{-2}K^{-1} is the Boltzmann constant. The last term stands for the transform of mechanical energy to thermal energy and describes heat dissipation of the stress wave [14, 15]. The temperature dependencies of lattice heat capacity and thermal conductivities are taken from Refs. [16, 17].

III. THERMO-ELASTOPLASTIC WAVE EQUATIONS

Thermoelastic deformation caused by nonuniform temperature distribution in glasses is determined by thermoelastic wave equations [18–20] as

$$\rho\frac{\partial^2\vec{u}}{\partial t^2} = G\nabla^2\vec{u} + \frac{G}{1-2\nu}\nabla(\nabla\cdot\vec{u}) - B\beta\nabla T_i, \tag{7}$$

where ν is Poisson coefficient, ρ is the material density, E is the longitudinal elastic modulus, $B = \frac{E}{3(1-2\nu)}$ is the bulk elastic modulus, and $G = \frac{E}{2(1+\nu)}$ is the shear elastic modulus. The temperature dependence of elastic moduli and Poisson coefficient is taken into account as it was proposed by Parc et al. [21] as follows

$$\begin{cases} E = (97 - \frac{1200}{T_i+1200}\cdot 24)\text{GPa} \\ B = (60 - \frac{1200}{T_i+1200}\cdot 23.2)\text{GPa} \\ G = (33.5 - \frac{1173}{T_i+1173}\cdot 2.3)\text{GPa} \\ \nu = 0.2 - \frac{1200}{T_i+1200}\cdot 0.03. \end{cases} \tag{8}$$

The density is calculated based on the continuity equation as follows

$$\frac{\partial\rho}{\partial t} + \nabla\cdot\left(\rho\frac{\partial\vec{u}}{\partial t}\right) = 0. \tag{9}$$

IV. CRITERION FOR CAVITATION

To define the conditions for cavitation inside fused silica bulk, the viscoelastic energy conservation law is used. According to the Grady's spall criterion for liquids [22], the sum of both elastic and kinetic energies should be greater than the surface energy, required to fracture the liquid into nanocavities of size R, plus the local viscous dissipation during void growth and coalescence in the cavitation process as follows

$$\frac{S_{dyn}^2}{4B} + \frac{\rho\zeta^2 R^2}{120} \geq \frac{6\sigma}{R} + \eta\zeta, \tag{10}$$

where S_{dyn} is the dynamic tensile strength required for cavitation, $\eta(T_i)$ is the viscosity, $\sigma(T_i) = \sigma_0(1 - T_i/T_{cr})^\alpha$ is the surface tension, $\sigma_0 = 0.3$ N/m is the surface tension constant [23], $\alpha = 1.25$ is the critical index [17], $\zeta = -\frac{\Delta\rho}{\rho\Delta t}$ is the strain rate, $\Delta\rho < 0$ is the density change, corresponding to material's expansion and rarefaction, Δt is the characteristic time of the deformation, and ρ is fused silica density. The first term $\frac{S_{dyn}^2}{4B}$ has the main contribution and expresses the elastic energy of deformation. The second term $\frac{\rho\zeta^2 R^2}{120}$ is the corresponding kinetic energy. The work against tension forces $\frac{6\sigma}{R}$ and dissipation forces $\eta\zeta$ are proportional to the surface tension $\sigma(T_i)$ and the viscosity $\eta(T_i)$, which both depend on the laser-induced temperatures in glass (decrease with the increasing temperature).

V. RAYLEIGH-PLESSET EQUATION

The Rayleigh-Plesset equation is then solved to analyze the nanopores dynamics [24, 25], which is written as follows

$$\ddot{R} = -\frac{3}{2}\frac{(\dot{R})^2}{R} - \frac{4\eta\dot{R}}{\rho R^2} - \frac{\Delta P}{\rho R} - \frac{2\sigma}{\rho R^2}, \tag{11}$$

where $R(t)$ is the characteristic size of nanopores, $\dot{R}$ and $\ddot{R}$ are the first and the second derivatives of the size function, $\Delta P \approx -\frac{3}{2}N_a k_B T_i$ is the negative pressure within the formed nanobubble. The initial conditions are set as $R(0) = R_0$ and $\dot{R}(0) = 0$, where R_0 is the initial size of nanopores. The pressure term $-\frac{\Delta P}{\rho R}$ has a positive contribution and, therefore, stands for the growth of the nanopores. The surface tension term $-\frac{2\sigma}{\rho R^2}$ and the term $-\frac{3}{2}\frac{(\dot{R})^2}{R}$ have a negative contribution and can result in the nanovoid collapse. Finally, the viscosity term $-\frac{4\eta\dot{R}}{\rho R^2}$ can be associated with the resistance to the material deformation, as it acts always against the direction of the nanovoid evolution $\dot{R}$.

The thermo-mechanical properties for fused silica and borosilicate glass used for the hydrodynamic criterion of the nanovoid growth are given in Table I.

Table I. Thermo-mechanical properties of glasses.

Physical properties	Fused silica	Borosilicate glass
Density $\rho[g/cm^3]$	2.2[26–28]	2.2[26, 27]
Heat capacity $C_i[J/(kgK)]$	1335-1440[17](1600-2400K)	830[26, 27]
Thermal conductivity $\kappa_i[W/(mK)]$	1.4-3.0[16](300-2500K)	1.1-1.2[26, 27]
Softening temperature T[K]	1875[27]	1093[27]
Thermal expansion $\beta \cdot 10^{-7}[1/K]$	5.5[28]	32.5[27]
Bulk modulus B[GPa]	35-44[21] (200-1800K)	35

[1] P. Velpula, M. Bhuyan, F. Courvoisier, H. Zhang, J. Colombier, and R. Stoian, Laser & Photonics Reviews **10**, 230 (2016).
[2] T. Itina, O. Uteza, N. Sanner, and M. Sentis, Journal of Optoelectronics and Advanced Materials **12**, 470 (2010).
[3] A. Rudenko, J.-P. Colombier, and T. E. Itina, Physical Review B **93**, 075427 (2016).
[4] A. Rudenko, J.-P. Colombier, and T. E. Itina, Int. J. Numer. Model. **e2215**, 1 (2016).
[5] L. Keldysh *et al.*, Sov. Phys. JETP **20**, 1307 (1965).
[6] A. Rudenko, J.-P. Colombier, and T. E. Itina, Journal of Laser Micro/Nanoengineering **11**, 304 (2016).
[7] B. Rethfeld, Phys. Rev. Lett. **92**, 187401 (2004).
[8] J. R. Gulley, Optical Engineering **51**, 121805 (2012).

[9] P. P. Rajeev, M. Gertsvolf, P. B. Corkum, and D. M. Rayner, Phys. Rev. Lett. **102**, 083001 (2009).
[10] M. Jupé, L. Jensen, A. Melninkaitis, V. Sirutkaitis, and D. Ristau, Opt. Express **17**, 12269 (2009).
[11] N. M. Bulgakova, V. P. Zhukov, and Y. P. Meshcheryakov, Applied Physics B **113**, 437 (2013).
[12] P. Martin, S. Guizard, P. Daguzan, G. Petite, P. D'oliveira, P. Meynadier, and M. Perdrix, Physical Review B **55**, 5799 (1997).
[13] N. M. Bulgakova, R. Stoian, and A. Rosenfeld, Quantum Electronics **40**, 966 (2010).
[14] X. Wang and X. Xu, Applied Physics A: Materials Science & Processing **73**, 107 (2001).
[15] Y. P. Meshcheryakov, M. V. Shugaev, T. Mattle, T. Lippert, and N. M. Bulgakova, Applied Physics A **113**, 521 (2013).
[16] P. Combis, P. Cormont, L. Gallais, D. Hebert, L. Robin, and J.-L. Rullier, Applied Physics Letters **101**, 211908 (2012).
[17] R. Kraus, S. Stewart, D. Swift, C. Bolme, R. Smith, S. Hamel, B. Hammel, D. Spaulding, D. Hicks, J. Eggert, *et al.*, Journal of Geophysical Research: Planets **117**, 1 (2012).
[18] D. Albagli, M. Dark, L. Perelman, C. Von Rosenberg, I. Itzkan, and M. Feld, Optics Letters **19**, 1684 (1994).
[19] I. Itzkan, D. Albagli, M. L. Dark, L. T. Perelman, C. von Rosenberg, and M. S. Feld, Proceedings of the National Academy of Sciences **92**, 1960 (1995).
[20] G. Paltauf and P. E. Dyer, Chemical reviews **103**, 487 (2003).
[21] R. L. Parc, C. Levelut, J. Pelous, V. Martinez, and B. Champagnon, Journal of Physics: Condensed Matter **18**, 7507 (2006).
[22] D. Grady, Journal of the Mechanics and Physics of Solids **36**, 353 (1988).
[23] K. Boyd, H. Ebendorff-Heidepriem, T. M. Monro, and J. Munch, Optical Materials Express **2**, 1101 (2012).
[24] M. Plesset, Journal of Applied Mechanics **16**, 277 (1949).
[25] A. Y. Kuksin, G. Norman, V. Pisarev, V. Stegailov, and A. Yanilkin, Physical Review B **82**, 174101 (2010).
[26] M. Lancry, F. Zimmerman, R. Desmarchelier, J. Tian, F. Brisset, S. Nolte, and B. Poumellec, Applied Physics B **122**, 66 (2016).
[27] S. Richter, F. Zimmermann, S. Döring, A. Tünnermann, and S. Nolte, Applied Physics A **110**, 9 (2013).
[28] Y. Okamoto, I. Miyamoto, K. Cvecek, A. Okada, K. Takahashi, and M. Schmidt, Journal of Laser Micro Nanoengineering **8**, 65 (2013).
